# Effects of Precipitates Evolution on Low Stress Creep Properties in P92 Heat-resistant Steel

**DOI:** 10.1038/s41598-018-33814-z

**Published:** 2018-10-18

**Authors:** Hongguang Han, Junjie Shen, Jiaxing Xie

**Affiliations:** 1 Tianjin Key Laboratory for Advanced Mechatronic System Design and Intelligent Control, School of Mechanical Engineering, Tianjin University of Technology, Tianjin, 300384 China; 2 National Demonstration Center for Experimental Mechanical and Electrical Engineering Education, Tianjin University of Technology, Tianjin, 300384 China

**Keywords:** Metals and alloys, Computational methods

## Abstract

The evolution of precipitates in P92 heat-resistant steel sustained under thermal aging at 923 K for different times were analyzed by scanning electron microscopy (SEM) and transmission electron microscopy (TEM), while the effects of precipitates evolution on the low stress creep properties were experimentally analyzed through uniaxial creep testing and multiaxial “helicoid spring creep testing”. The results demonstrate that the coarsening of M_23_C_6_ carbide is significantly slower than the Laves phase in the thermal aging of 0~8000 h. The creep resistance of the P92 heat-resistant steel is enhanced at 923 K aging for 3000 h, and the strengthening effect is significantly apparent under the lower stresses. Moreover, the microstructure degradation factor of the P92 heat-resistant steel in the low stress region at 923 K is different that under the lower stresses of 20 MPa and 35 MPa is mainly Laves phase and the higher stresses of 65 MPa and 75 MPa is mainly M_23_C_6_ carbide.

## Introduction

Ferritic heat-resistant steels containing 9~12% Cr have been widely utilized for high temperature and high pressure parts of ultra-supercritical thermal power units due to the corresponding outstanding creep resistance, good corrosion resistance and desirable high temperature oxidation resistance. The high-temperature creep rupture failure of these heat-resistant steels will directly cause severe impact on the safety and normal production of the thermal power plants. Nowadays the creep rupture life of these heat-resistant steels is generally predicted through the time-temperature parameter method based on high-stress creep data in engineering applications^1^. However, the research results demonstrated that the predicted low-stress creep rupture life of these heat-resistant steels through this method was higher than the measured value^2–4^, namely the creep resistance property in the low-stress region was degraded.

The creep performance degradation of ferritic heat-resistant steels containing 9~12% Cr in the low-stress region can be attributed to the reduction of obstacles inhibiting dislocation movement, which is caused by the coarsening of precipitates and the annihilation of subboundaries in the heat-resistant steels under the long-term high temperature aging^5,6^. Hald^7^ and Abe^8^ considered that the ferritic heat-resistant steels containing 9~12% Cr were mainly strengthened by the inhibiting effects of fine and dispersed second phases precipitated from the subboundaries as well as the crystalline grains on the movement of dislocations and subboundaries. These inhibiting effects could delay the accumulation of creep strain, consequently leading in the strengthening effects achievement under long-term loading at high temperatures. Therefore, the creep resistance property of these steels was closely related to the precipitates evolution. The precipitates in the heat-resistant steels containing 9~12% Cr mainly include M_23_C_6_ carbides, Laves phases, MX phases and Z phases^9–11^. The MX phases retain relatively higher stability and their sizes remain unchanged during long-term thermal aging^9,11^; in contrast, the M_23_C_6_ carbides and Laves phases apparently increase in size at the original austenite grain boundaries as well as the subboundaries^12^. Although the precipitation of Laves phases can compensate the degradation of creep-rupture properties caused by the weakening of solid solution strengthening effects from W and Mo atoms in a relatively short time, but the aggregation and coarsening of Laves phases accompanied with the long-term thermal aging and creep can lead to the creep-rupture performance degradation^13,14^. The M_23_C_6_ carbide is an important factor that maintains the lath structure stability of the tempered martensite in high Cr ferritic heat-resistant steels^15,16^. Differently from the short-time precipitation characteristics of all M_23_C_6_ carbides, MX phases and Laves phases, the precipitation of Z phases generally requires a relatively long thermal aging time. For example, the Z phases begin to precipitate after thermal aging at 923 K for 20000 h in 9% Cr steels^10^. The precipitation of Z phases can consume the beneficial MX phases in the matrix and consequently lead to the premature degradation of the long-term creep rupture performance^17^. Yoshizawa *et al*.^18^ considered that Z phases were not a necessary factor that caused the degradation of creep rupture performance. Nevertheless, the latter investigation regarding the effects of precipitates on the creep rupture performance was entirely built based on the short-term high-stress accelerated creep experiments. Related studies have demonstrated that the creep properties of heat-resistant steels differ in the high stress and low stress regions^3^.

It is extremely difficult to perform uniaxial long-term creep tensile tests under extremely low stresses and further explore the effects of precipitate evolutions on the low stress creep property. This is because the uniaxial creep experiment based on axial tension has a low strain resolution under extremely low stresses, which cannot meet the experimental requirements. In view of this, the multiaxial “helicoid spring creep experiment method” based on pure torsion was proposed and utilized to study the short-term creep behaviors of metal materials under extremely low stresses^19–21^. In this work, the P92 heat-resistant steel was selected as the research object. Thermal aging treatments at 923 K for 1500 h, 3000 h, 4000 h, 5500 h and 8000 h were conducted for various specimens. The precipitate type and the morphology characteristic analyzed with a scanning electron microscope (SEM) and a transmission electron microscope (TEM). Moreover, the short-term creep behaviors of the specimens treated for different aging times were obtained through the traditional rectangular creep and the helicoid spring creep experimentation methods. On this basis, the effects of precipitates evolution on the low stress creep property were analyzed, while the “microstructure degradation factor” was clarified.

## Results and Discussion

### Microstructure evolution

**Figure 1:**
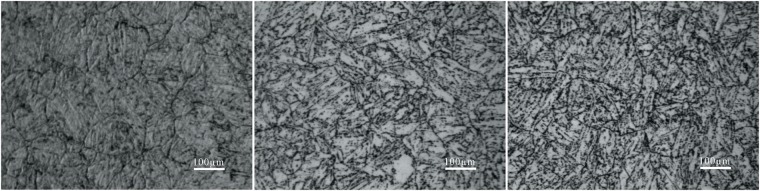
OM micrographs of P92 heat-resistant steels without aging (**a**) and following thermal aging for 3000 h (**b**) and 8000 h (**c**).

**Figure 2:**
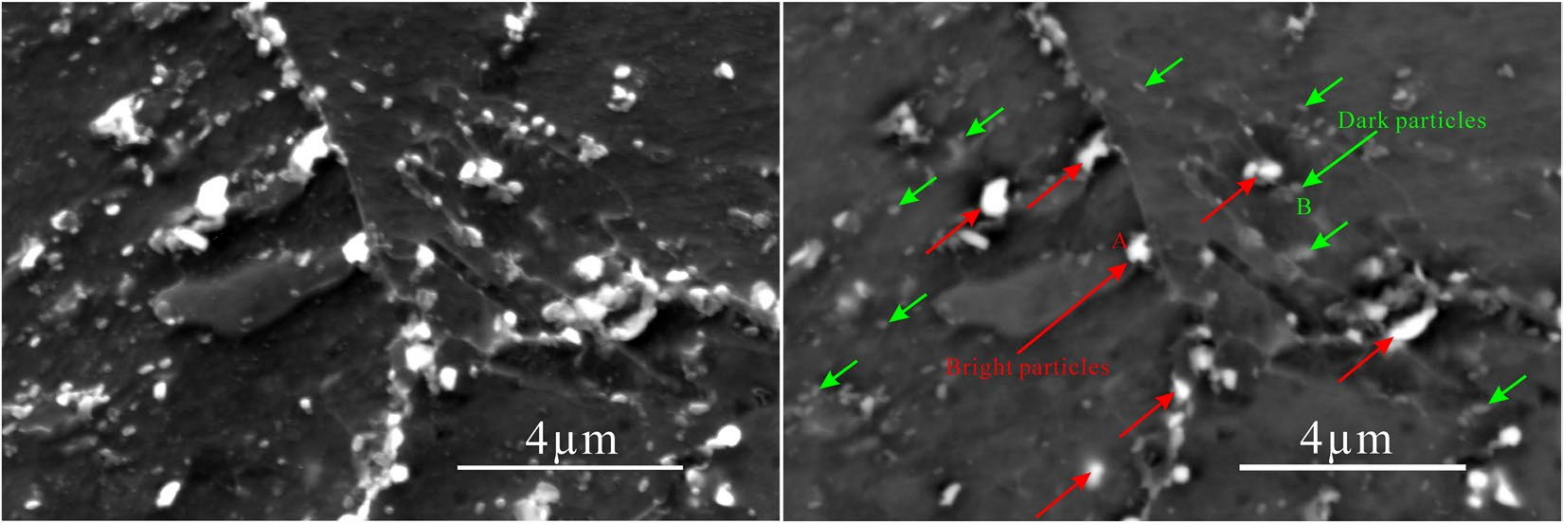
SEM (**a**) and SEM-BSE (**b**) micrographs of P92 heat-resistant steels following thermal aging for 3000 h.

**Figure 3:**
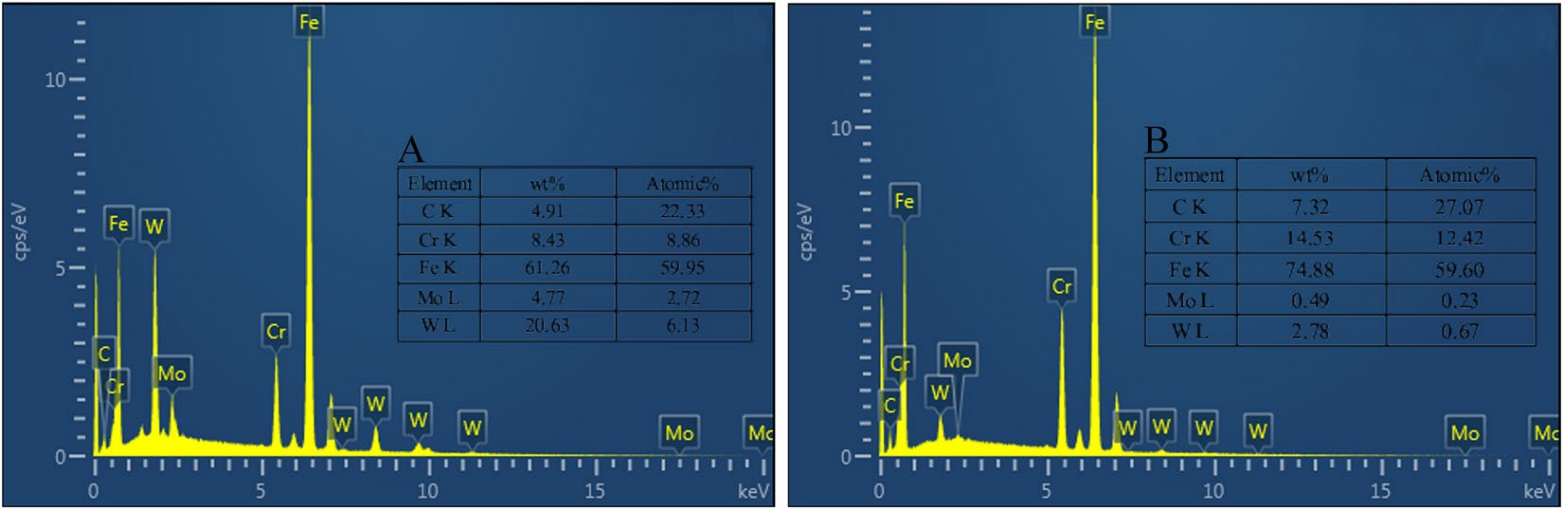
SEM-EDS diagrams of bright particles A (**a**) and dark particles B (**b**) in P92 heat-resistant steels following thermal aging for 3000 h.

**Figure 4:**
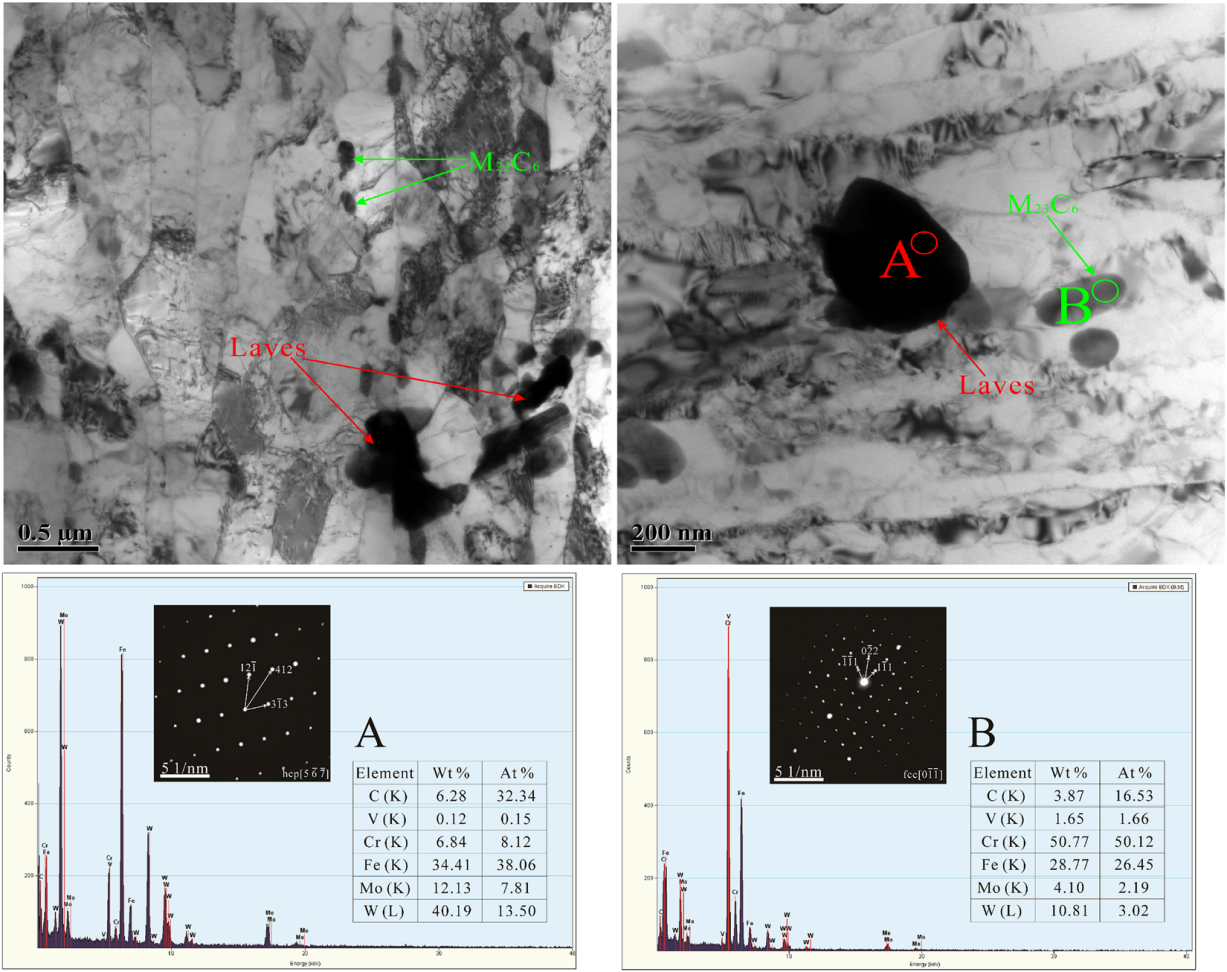
TEM micrographs of P92 heat-resistant steels following thermal aging for 3000 h (**a,b**) and the corresponding SAED patterns and EDS diagrams of particles A (**c**) and B (**d**).

**Figure 5:**
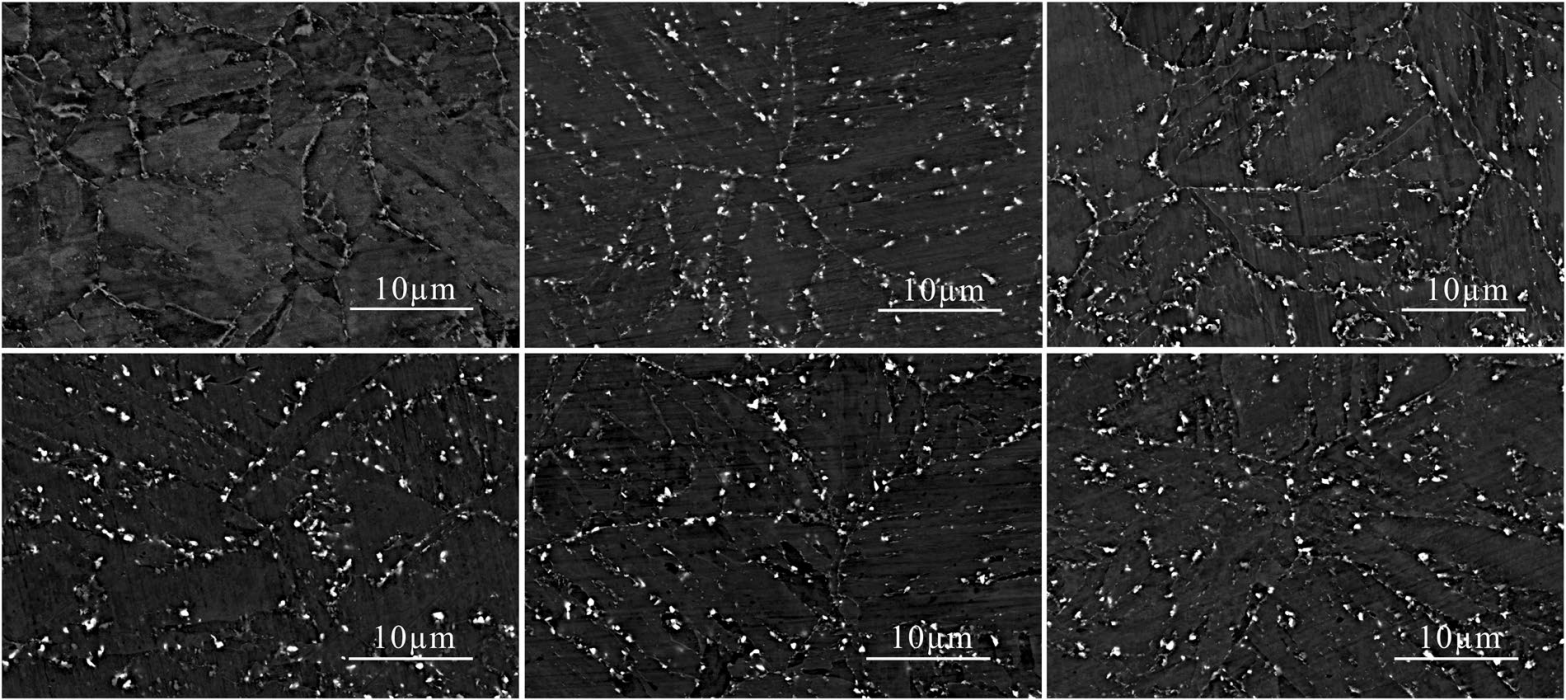
SEM-BSE micrographs of P92 heat-resistant steels following thermal aging for 0 h (**a**), 1500 h (**b**), 3000 h (**c**), 4000 h (**d**), 5500 h (**e**), 8000 h (**f**).

Figure 1 shows the OM images of the P92 heat-resistant steel subsequently to thermal aging for different times. The microstructure is typical tempered martensite which containing microstructure units such as lath-shaped martensite beams and blocks, and many precipitates are distributed in the matrix. It could be observed from Fig. 2(a,b) that two types of bright and dark shades of precipitates with various sizes are existed. The SEM-EDS analysis in Fig. 3(a,b) demonstrates that the brightest precipitates are rich in W and Fe, the dark precipitates are rich in Fe and Cr. Figure 4(a,b) presents the TEM images of P92 heat-resistant steels following thermal aging for 3000 h, typical tempered martensite can be identified clearly. Furthermore, two types of precipitates are observed. One is a small size rod-like particle B. The other is a larger size irregular shape particle A. Selected area electron diffraction (TEM-SAED) analysis in Fig. 4(c,d) shows that the crystal structure of particle A is close packed hexagonal, and the crystal structure of particle B is face-centered cube. TEM-EDS analysis from Fig. 4(c,d) shows that particle A is rich in W and Fe which is inferred to be Fe_2_W-type Laves phase, while particle B is rich in Cr which is presumed to be Cr_23_C_6_-type M_23_C_6_ carbide. Therefore, it could be determined that these two types of bright and dark shades of precipitates in the BSE image were Laves phases and M_23_C_6_ carbides respectively. Figure 5(a–f) presents the SEM-BSE images of P92 heat-resistant steels following thermal aging for 0 h (unaged) ~8000 h respectively. It could be observed that the M_23_C_6_ carbides existed in the specimen without thermal aging, which mainly distributed at the grain boundaries and subboundaries of the original austenite. The Laves phases only existed in the specimens following thermal aging, which mainly distributed at the interface of the original austenite grain boundary and the lath-shaped phase boundary, as well as the vicinity of the M_23_C_6_ carbides.

**Figure 6:**
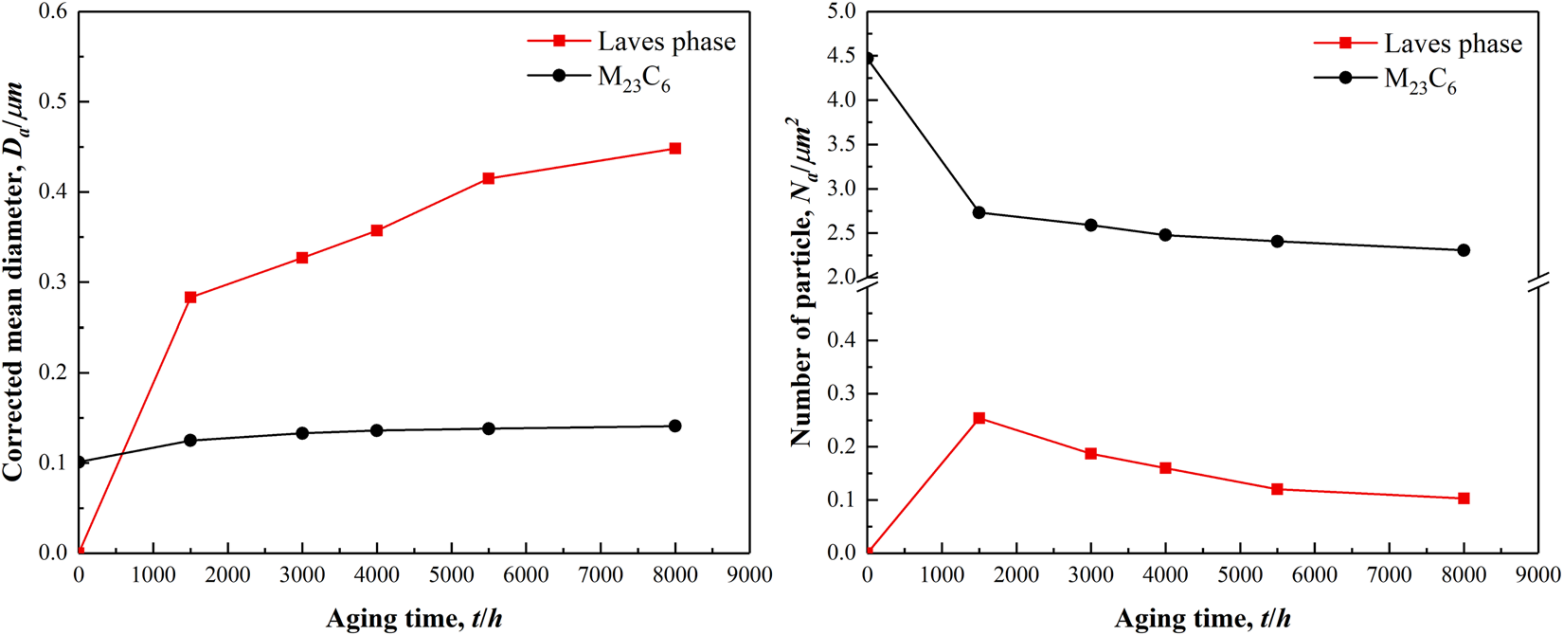
Average diameter of precipitates (**a**) and the number of precipitates per unit area (**b**) of M_23_C_6_ carbides and Laves phases in P92 heat-resistant steels following thermal aging for different times.

To investigate the average diameter of precipitates and the number of precipitates per unit area, the Image-Pro Plus software was utilized to measure the number of precipitates per unit area and the average diameter of precipitates in the SEM-BSE images. Subsequently, the measured average diameter values were corrected. This is because the measured particle diameter of precipitates is not the actual average diameter of the precipitates^22^. The corrected average diameter of the precipitates is *D*_*a*_ = 4*D*_*ob*_ /π, where *D*_*ob*_ is the average diameter of the precipitates. It could be observed from Fig. 6a that the M_23_C_6_ carbides increased in size slightly which increased from 100 nm without thermal aging to 141 nm with thermal aging for 8000 h. However, the diameters of the Laves phases without aging and following thermal aging for 3000 h and 8000 h are 0 nm, 327 nm and 448 nm respectively, which means the variation of overall size is quite apparent. Figure 6b shows the number of M_23_C_6_ carbides per unit area decreased relatively faster following thermal aging for 1500 h, consequently decreasing slowly, while the number of Laves phases per unit area reached the maximum value of 0.254/µm^2^ in thermal aging for 1500 h, then decreased and tended to a stable value. These results demonstrate that the coarsening rate of the Laves phases is significantly higher than the M_23_C_6_ carbides during thermal aging, which is consistent with the results of quantitative analysis by Hättestrand *et al*.^23^.

### Short-term creep behaviors

**Figure 7:**
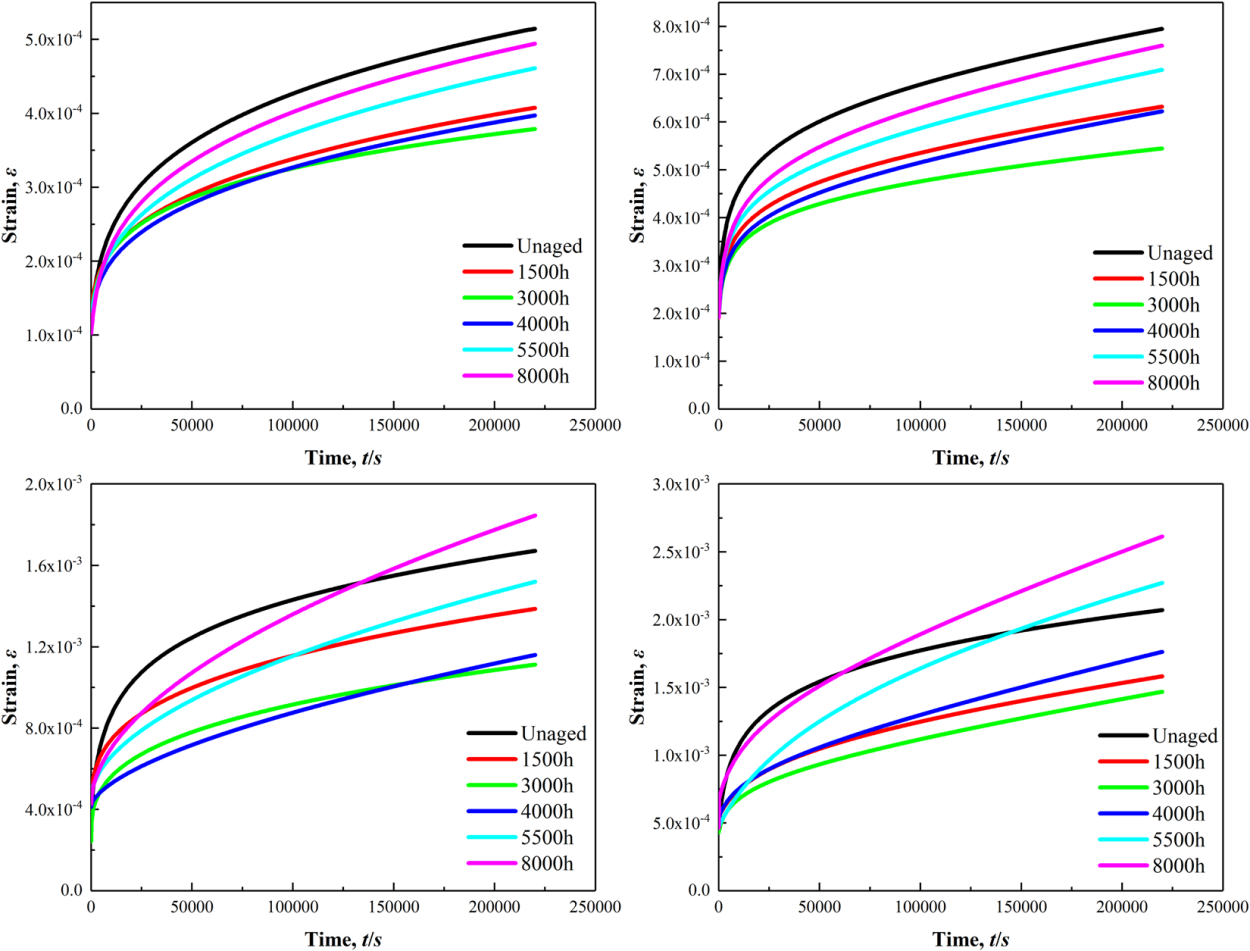
Creep curves of P92 heat-resistant steel specimens following thermal aging for different times at 923 K under stress of 20 MPa (**a**), 35 MPa (**b**), 65 MPa (**c**) and 75 MPa (**d**).

**Figure 8:**
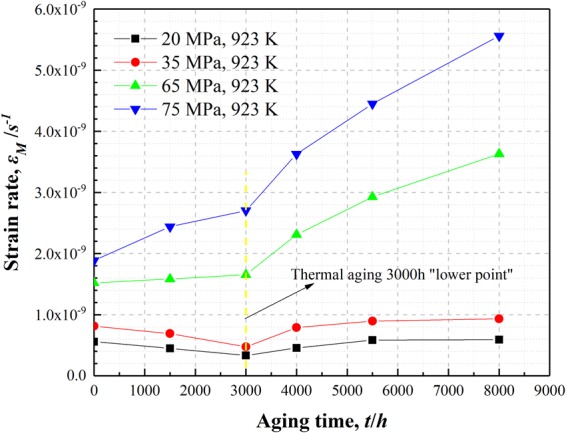
Creep rate *ε*_*M*_ curves of P92 heat-resistant steel specimens following thermal aging for different times at 923 K under stress of 20 MPa, 35 MPa, 65 MPa and 75 MPa.

Figure 7 shows the creep curves of P92 heat-resistant steel for different thermal aging times at 923 K under the stress of 20 MPa, 35 MPa, 65 MPa and 75 MPa. The creep strain amount gradually increased, whereas the creep rate gradually decreased and tended to an approximate stability level until 220 Ks as the creep time increased. It is discovered that the specimens after thermal aging for 3000 h had the minimum total strain amount under the stress of 20 MPa, 35 MPa, 65 MPa and 75 MPa until the short-term creep experimentation is completed. In this paper, the Origin Pro software was utilized to obtain the creep rate *ε*_*M*_ that at the end of the short-term creep experimentation. The creep rate *ε*_*M*_ was used to characterize the microstructure evolution effects on the low stress creep resistance property. Figure 8 presents that the creep rate *ε*_*M*_ at 923 K decreased gradually under the stress of 20 MPa and 35 MPa from thermal aging 0 h to 3000 h, while it presents a gradually increasing trend under the stress of 65 MPa and 75 MPa. The creep rate *ε*_*M*_ under the stress values of 20 MPa, 35 MPa, 65 MPa and 75 MPa are all presented an increasing trend following thermal aging for 3000 h~8000 h. However, the increasing trend of the creep rate at 923 K under the stress of 20 MPa and 35 MPa for the specimens following thermal aging for 3000~5500 h slows down and the creep rate even decreased slightly for the specimens subsequently to thermal aging for 5500~8000 h. By contrast, the creep rate *ε*_*M*_ at 923 K under the stress of 65 MPa and 75 MPa always presents an increasing trend, while the increasing trend slightly slow down for the specimens following thermal aging for 5500~8000 h.

### Effects of microstructure evolution on the low stress creep property

The ferritic heat-resistant steels containing 9~12% Cr are mainly strengthened by the inhibiting effects of fine and dispersed second phases, precipitated from the subboundaries and crystalline grains on the movement of dislocations and subboundaries, which can delay the accumulation of creep strain and enhance the strengthening effects under long-term loading at high temperatures^7,8^. Adversely, during the long-term high-temperature thermal aging and in the creep testing, the coarsening of precipitates and the annihilation of subboundaries in the heat-resistant steels eventually lead to the degradation of the low stress creep property of the heat-resistant steels^5,6^. Consequently, the effects of precipitates evolution on the creep performance in the low stress region were emphatically analyzed among the much influencing factors on the long-term creep performance. Through the variation trends comparison of the creep rate *ε*_*M*_ at 923 K under different stresses, it could be observed that lower point of strain rate decrease occurred in the variation trend curves of the overall creep rate *ε*_*M*_ for the specimens following thermal aging for 3000 h (as shown by the yellow dotted line in Fig. 8). Moreover, Fig. 7 shows that the creep curves of the specimen after thermal aging for 3000 h under the stress of 20 MPa, 35 MPa, 65 MPa and 75 MPa are the lowest creep curves. These results indicate that the precipitates had certain strengthening effects on the creep resistance property of the short-term thermally aged specimens under different stresses, and the precipitates had more significantly strengthening effects under the relatively lower stress. Furthermore, from the variation trend of the creep rate *ε*_*M*_ for the specimens following thermal aging for 3000 h~8000 h, it could be observed that the creep rate *ε*_*M*_ presented an increasing trend under different stresses, but the increasing trend slowed down to a certain extent for the specimen following thermal aging for 8000 h. The difference is that the slow-down trend of the creep rate *ε*_*M*_ under the stress of 20 MPa and 35 MPa is more apparent than under the stress of 65 MPa and 75 MPa. Therefore, it should distinguish the difference whether the stress is high or low when considering the effects of precipitates on the low stress creep properties in P92 heat-resistant steel, which might relate to the contents of various precipitated phases. The equation that describes the effects of precipitates on the creep resistance property is similar to the Orowan stress equation^24^:

1 $$\sigma =3.32Gb\frac{\sqrt{{f}_{p}}}{{d}_{p}}$$

where *G* is the shear modulus (923 K, 64 Gpa), *b* is the modulus length of the Burgers vector (0.25 nm), *f*_*p*_ is the volume fraction of the precipitates and *d*_*p*_ is the average particle diameter^25^. The above formula presents that the content of precipitates directly affects the Orowan stress. During thermal aging at 923 K, the M_23_C_6_ precipitates content was higher compared to the Laves phases^23,26^. Wang *et al*.^25^ analyzed the Orowan stress generated from the M_23_C_6_ carbides and Laves phases during thermal aging at 923 K, discovering that the M_23_C_6_ carbides could provide higher Orowan stress values than the Laves phases. Moreover, the size and the content of Laves phases and M_23_C_6_ carbides are changing during thermal aging, which means the Orowan stress provided by the Laves phases and M_23_C_6_ carbides is varied.

**Figure 9:**
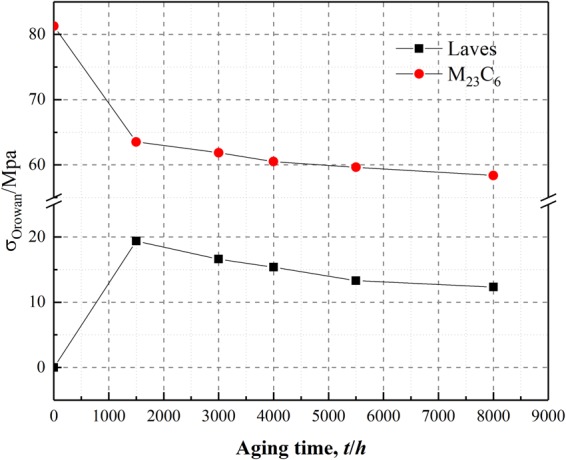
The Orowan stress produced by Laves phases and M_23_C_6_ carbides in P92 heat-resistant steel after 923 K different thermal aging.

Figure 9 shows that the Orowan stress produced by M_23_C_6_ carbides after different thermal aging was greater than the Orowan stress produced by Laves phases, which is consistent with the research of Wang *et al*.^25^. When the applied stresses are 20 MPa and 35 MPa, the applied stress is lower than the Orowan stress produced by the M_23_C_6_ carbides during thermal aging, so the dislocations could shear the M_23_C_6_ carbides mainly in the climbing mode during creep movement^27,28^. In contrast, when the applied stress is higher than the Orowan stress produced by the Laves phases during thermal aging, the dislocations would bypass the Laves phase particles according to the Orowan mechanism during creep movement^27^. When the applied stress is 65 MPa and 75 MPa, the Orowan stress provided by the M_23_C_6_ carbides and Laves phases is lower than the applied stress in both cases except Orowan stress of M_23_C_6_ carbides in unaged, so the dislocations would bypass the M_23_C_6_ carbides and Laves phases according to the Orowan mechanism during creep movement^27^. Regardless of how the dislocations passing the second phase particles to produce deformation, the precipitated phases always have inhibiting effects on the dislocation movement in the low stress creep regime. The difference is that the contribution to deformation when the dislocations bypass the second phase particles according to the Orowan mechanism is higher than when the dislocations shear the second phase particles in the climbing mode. Therefore, the microstructure degradation factor that affects the low stress creep at 923 K under 20 MPa and 35 MPa is probably the Laves phase. Because of the MX phase is relatively stable during long-term high-temperature aging^23^ and the Z phase could precipitate only after thermal aging for 20000 h at 923 K^10^. Therefore, in this paper, the effects of the MX and Z phases were not considered.

Actually, the creep rate *ε*_*M*_ of the specimen subsequently to thermal aging for 3000 h is the lowest in the creep experiments at 923 K under 20 MPa and 35 MPa, which means that the corresponding Orowan stress should be the highest. However, it can be seen from Fig. 9 that the Orowan stress value provided by the Laves phases is maximal at 1500 h aging. This might due to the dislocations gliding mechanism mainly in the mode of diffusion creep that make it can bypass the second phase particles under extremely low stress or higher temperatures. So another Orowan stress formula was introduced for analysis. The Orowan stress required by the dislocations for bypassing the particles is^29^:

2 $$\tau =\frac{Gb}{\lambda }$$

Where *G* is the shear modulus, *b* is the modulus length of the Burgers vector and *λ* is the space between the second phase particles. It is worth noting that the *λ* is inversely proportional to the number of particles in dislocation line per unit length. Assuming that the second phase particles are evenly distributed, the higher the number of precipitates is the lower the space between the second phase particles is, namely the higher the required Orowan stress for the particles being bypassed, which means that the macroscopic creep resistance property of the steel is enhanced. However, in this study, the specimen following thermal aging for 1500 h has the highest number density of Laves phase particles, but it did not possess the best creep resistance property. This indicates that the space between the second phase particles may also be related to the diameter of the precipitates. In fact, the smaller the size of the precipitates, the higher the number density and the better the strengthening effect. On these bases, the corresponding relationship between the precipitate parameters and the creep rate *ε*_*M*_ is attempted to be established. The following relationship through the numerical analysis of the precipitate parameters and the creep rate *ε*_*M*_ is obtained:

3 $${\varepsilon }_{M}\propto {H}_{s}={N}_{a}{D}_{a}^{2}$$

**Figure 10:**
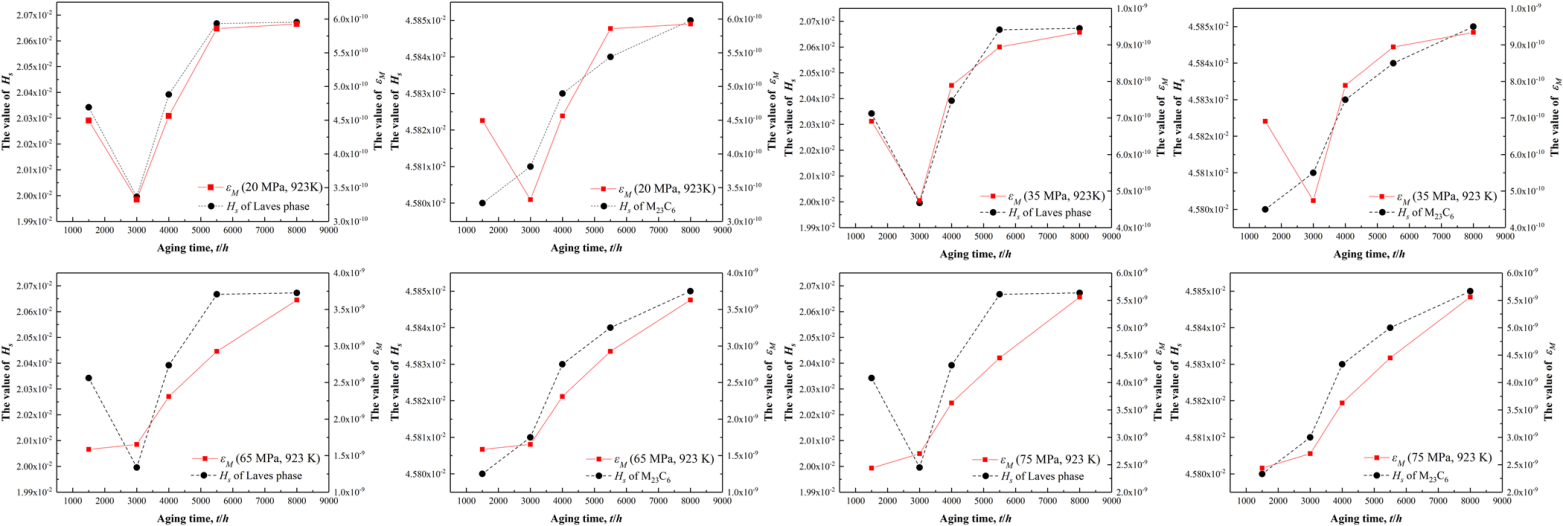
Comparison between the variation trends of *ε*_*M*_ values for P92 heat-resistant steel at 923 K under different stresses (20 MPa (**a,b**), 35 MPa (**c,d**), 65 MPa (**e,f**) and 75 MPa (**g,h**)) and *H*_*s*_ values for Laves phases and M_23_C_6_ carbides.

Where *N*_*a*_ is the number of precipitates per unit area (unit, *μm*^2^); *D*_*a*_ is the average diameter of precipitates (unit, *μm*); ***H***_s_is a dimensionless parameter that is related to the *N*_*a*_ and *D*_*a*_ which reflects the precipitates evolution. Since Laves phases is not existed in the specimen without thermal aging, it is reasonable to investigate the effects of precipitates on the creep property in P92 heat-resistant steels with different thermal aging times under different stresses except the specimen without thermal aging. Through comparison between the *H*_*s*_ values after thermal aging for 1500 h~8000 h (as shown in Table 1) and the *ε*_*M*_ values under different stresses, it could be observed that the variation trend of the *H*_*s*_ values for the Laves phases is consistent with the *ε*_*M*_ values variation at 923 K under 20 MPa and 35 MPa (as shown in Fig. 10(a–d)), whereas the variation trend of *H*_*s*_ values for the M_23_C_6_ carbide is consistent with the *ε*_*M*_ values variation trend at 923 K under 65 MPa and 75 MPa (as shown in Fig. 10(e–h)). Yamasaki *et al*. discovered that the creep property of P92 ferritic heat-resistant steel also broke over at 923 K under the tensile stress of approximately 60 MPa^30^. This further demonstrates that the creep resistance properties of P92 ferritic heat-resistant steel at 923 K differed under different stresses in the low-stress region. Therefore, the precipitates which affect the creep property of the P92 ferritic heat-resistant steels under the stress of 20 MPa and 35 MPa at 923 K is mainly the Laves phases, while the precipitates which affect the creep property of the P92 ferritic heat-resistant steels under the stress of 65 MPa and 75 MPa at 923 K is mainly the M_23_C_6_ carbides.

**Table 1:** *H*_*s*_ values of M_23_C_6_ carbides and Laves phases in P92 heat-resistant steels following thermal aging for different times.

Precipitates	Time(*h*)	*N*_*a*_(/*µm*^*−2*^)	*D*_*a*_(*µm*)	*Hs*
Laves phases	1500	0.254	0.283	0.020342606
	3000	0.187	0.327	0.019995723
	4000	0.160	0.357	0.020391840
	5500	0.120	0.415	0.020667001
	8000	0.103	0.448	0.020672512
M_23_C_6_ carbides	1500	2.731	0.125	0.045796875
	3000	2.590	0.133	0.045814510
	4000	2.478	0.136	0.045833088
	5500	2.407	0.138	0.045838908
	8000	2.306	0.141	0.045845586

## Methods

**Figure 11:**
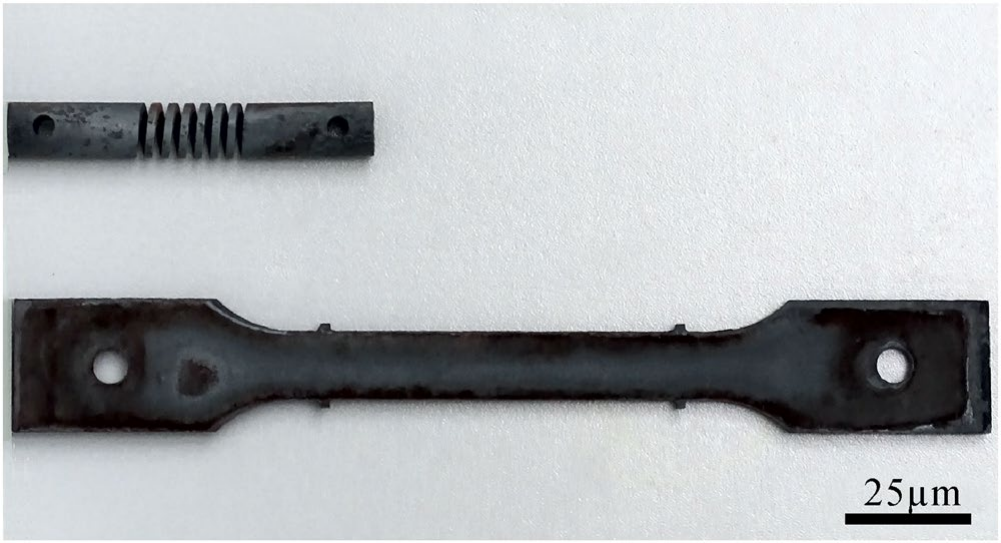
Helical spring and rectangular specimens following thermal treatment.

The ASME Grade P92 steel was utilized in the experiments and its chemical composition could be observed in Table 2. The heat treatments were as follows: normalizing at 1323 K for 1 h + air cooling, tempering at 1033 K for 1 h + air cooling (without aging), thermal aging at 923 K for 1500 h, 3000 h, 4000 h, 5500 h and 8000 h for various specimens. Cubic specimens of 5 mm × 5 mm × 5 mm were cut for microstructural observations. Helical spring specimens and traditional rectangular specimens (as presented in Fig. 11) were prepared through mechanical machining. The outer and inner diameters of the helical spring is 12 mm and 8 mm respectively, while the cross section of the helicoid coil is a rectangle of 2 mm × 2 mm in dimensions. The rectangular specimen is of 220 mm in length, of 2 mm in thickness and of 15 mm in width in the middle part; its surface roughness subsequently to machining is 1.6 *µm*.

**Table 2:** Chemical composition of Grade P92 steel (wt. %).

C	Si	Mn	S	P	Cr	Mo	W	V	Nb	Ni	N	B
0.094	0.120	0.480	0.003	0.009	9.230	0.410	1.790	0.200	0.054	0.160	0.043	0.003

Firstly, the cubic specimens subsequently to thermal aging for different times were ground with diamond sand papers and polished with the polishing agent. Following, the Vilella’s reagent (main ingredients were 1 g of picrate, 5 mL of hydrochloric acid and 100 mL of ethanol) was utilized for the surface corrosion of these specimens. The samples for TEM observation were ground to about 50 *μm* with diamond sand papers, and polished with the polishing agent. Then it was thinned by electrolysis double-spray thinning instrument. The macroscopic morphology of the thermally aged specimens was observed with an optical microscope (OM, OLYMPUS-BX51M). The microstructure of the specimen was observed with a transmission electron microscope (TEM, FEI Tecnai F30) and a scanning electron microscope ((SEM, Quanta FEG 250). The images of multiple regions of the thermally aged specimens were collected for the quantitative analysis of the precipitates. Furthermore, the compositions of the precipitates were identified through SEM-EDS and TEM-EDS, and selected area electron diffraction of the transmission electron microscope (TEM-SAED) was used to further identify the precipitates type.

**Figure 12:**
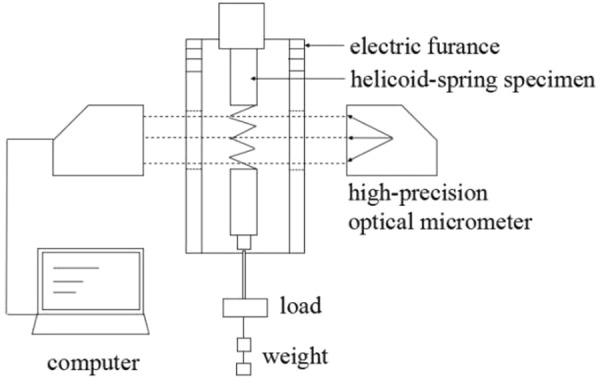
Helicoid spring creep apparatus under lower stresses including electric furnace, high-precision optical micrometer, load, weight, as well as information receiving and processing system.

According to the research work of Kimura *et al*.^3^, the relationship between the minimum creep rate and stress at 923 K broke over under the tensile stress of approximately 90 MPa. Recently, it was also indicated that the creep resistance property of the heat-resistant steels varied under the tensile stress of approximately 60 MPa in the low stress region^30^. Therefore, tensile stresses of 20 MPa, 35 MPa, 65 MPa and 75 MPa in the low stress region were adopted. With consideration to the low strain resolution of the traditional uniaxial creep experiment, the helicoid spring creep experimental method was utilized to conduct the creep experiments at 923 K under the load of 20 MPa and 35 MPa^31–34^. The helicoid spring creep equipment is presented in Fig. 12. In this study, the value of coil-pitch spacing is between 2.0 mm to 4.0 mm. Therefore, torsion is the dominant component of deformation in the helicoid spring creep experimental method^35^. Since the stress or strain of the helicoid spring is essentially shear components, they can be converted into equivalent parameters through the Von-Mises equation. At the temperature of 923 K, under the load of 65 MPa and 75 MPa, the creep experiments were conducted through the traditional uniaxial rectangular creep experimentation method. All specimens for creep experiments sustained thermal aging treatment for 0 h, 1500 h, 3000 h, 4000 h, 5500 h and 8000 h respectively, whereas the time for the short-term creep experiments was 220 Ks.

## Conclusions

The precipitates evolution was studied through thermal aging for different times in the P92 heat-resistant steel specimens, while the evolution of the precipitates were analyzed by scanning electron microscopy (SEM) and transmission electron microscopy (TEM). The effects of precipitates evolution on the low stress creep properties were experimentally analyzed through uniaxial creep testing and multiaxial “helicoid spring creep testing”. Through the comparison between the ${H}_{s}\,$values that reflects the precipitates evolution and the *ε*_*M*_ values in the end of the short-term creep experiment that reflected the creep property, the microstructure degradation factor under different stresses was obtained. The specific research conclusions could be drawn as follows:

1. During thermal aging in P92 heat-resistant steel at 923 K, the Laves phases could precipitate only subsequently to thermal aging, whereas the M_23_C_6_ carbide existed in the specimens prior to thermal aging. Moreover, the coarsening rate of M_23_C_6_ carbide is significantly lower than the Laves phase.
2. The creep property of the P92 heat-resistant steel specimens following thermal aging at 923 K for 3000 h is enhanced under different stresses, and the strengthening effects under the lower stress of 20 MPa and 35 MPa are quite significant.
3. The microstructure degradation factor of the P92 heat-resistant steel in the low stress region at 923 K is different that under the lower stresses of 20 MPa and 35 MPa is mainly Laves phase and the higher stresses of 65 MPa and 75 MPa is mainly M_23_C_6_ carbide.
