# Author Correction: Effects of Precipitates Evolution on Low Stress Creep Properties in P92 Heat-resistant Steel

**DOI:** 10.1038/s41598-019-41875-x

**Published:** 2019-06-27

**Authors:** Hongguang Han, Junjie Shen, Jiaxing Xie

**Affiliations:** 1 Tianjin Key Laboratory for Advanced Mechatronic System Design and Intelligent Control, School of Mechanical Engineering, Tianjin University of Technology, Tianjin, 300384 China; 2 National Demonstration Center for Experimental Mechanical and Electrical Engineering Education, Tianjin University of Technology, Tianjin, 300384 China

Correction to: *Scientific Reports* 10.1038/s41598-018-33814-z, published online 18 October 2018

This Article contains errors in Figures 1, 2, 3, 4, 5, 6, 7 and 10, where images are unlabelled. The correct Figures 1, 2, 3, 4, 5, 6, 7 and 10 appear below as Figures 1–8 respectively.

**Figure 1:**
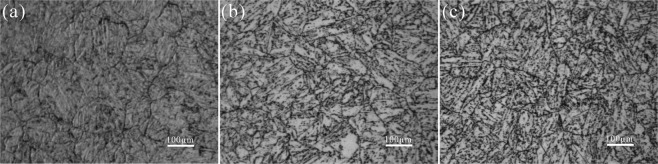
OM micrographs of P92 heat-resistant steels without aging (**a**) and following thermal aging for 3000 h (**b**) and 8000 h (**c**).

**Figure 2:**
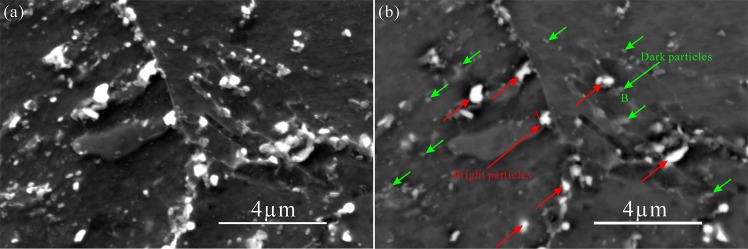
SEM (**a**) and SEM-BSE (**b**) micrographs of P92 heat-resistant steels following thermal aging for 3000 h.

**Figure 3:**
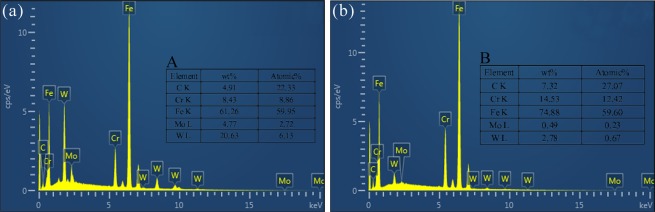
SEM-EDS diagrams of bright particles A (**a**) and dark particles B (**b**) in P92 heat-resistant steels following thermal aging for 3000 h.

**Figure 4:**
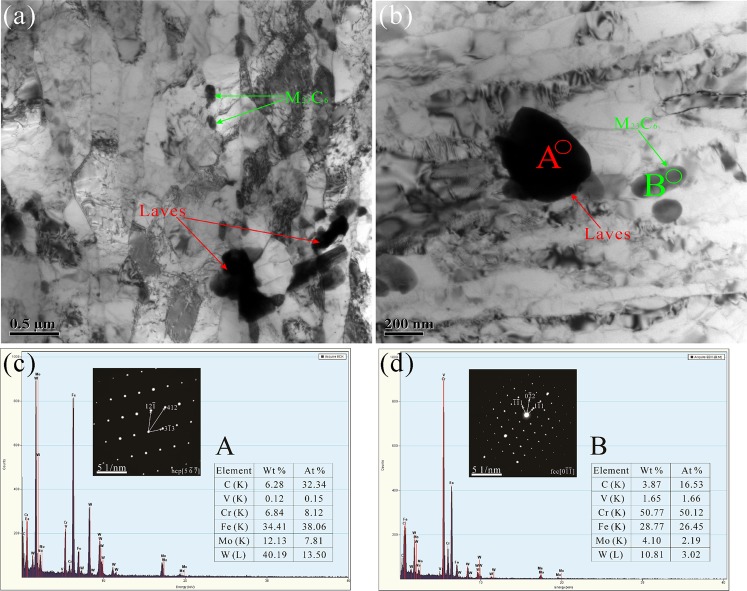
TEM micrographs of P92 heat-resistant steels following thermal aging for 3000 h (**a**,**b**) and the corresponding SAED patterns and EDS diagrams of particles A (**c**) and B (**d**).

**Figure 5:**
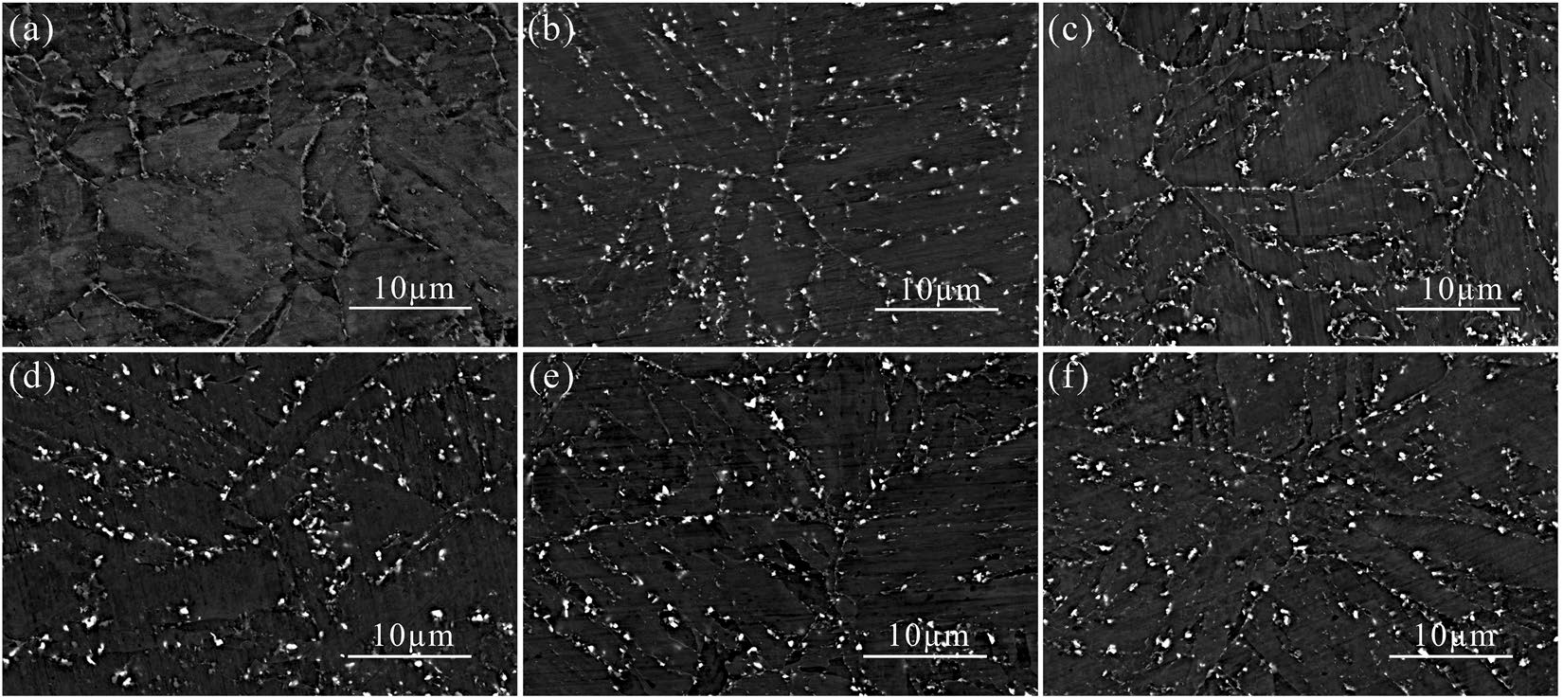
SEM-BSE micrographs of P92 heat-resistant steels following thermal aging for 0 h (**a**), 1500 h (**b**), 3000 h (**c**), 4000 h (**d**), 5500 h (**e**), 8000 h (**f**).

**Figure 6:**
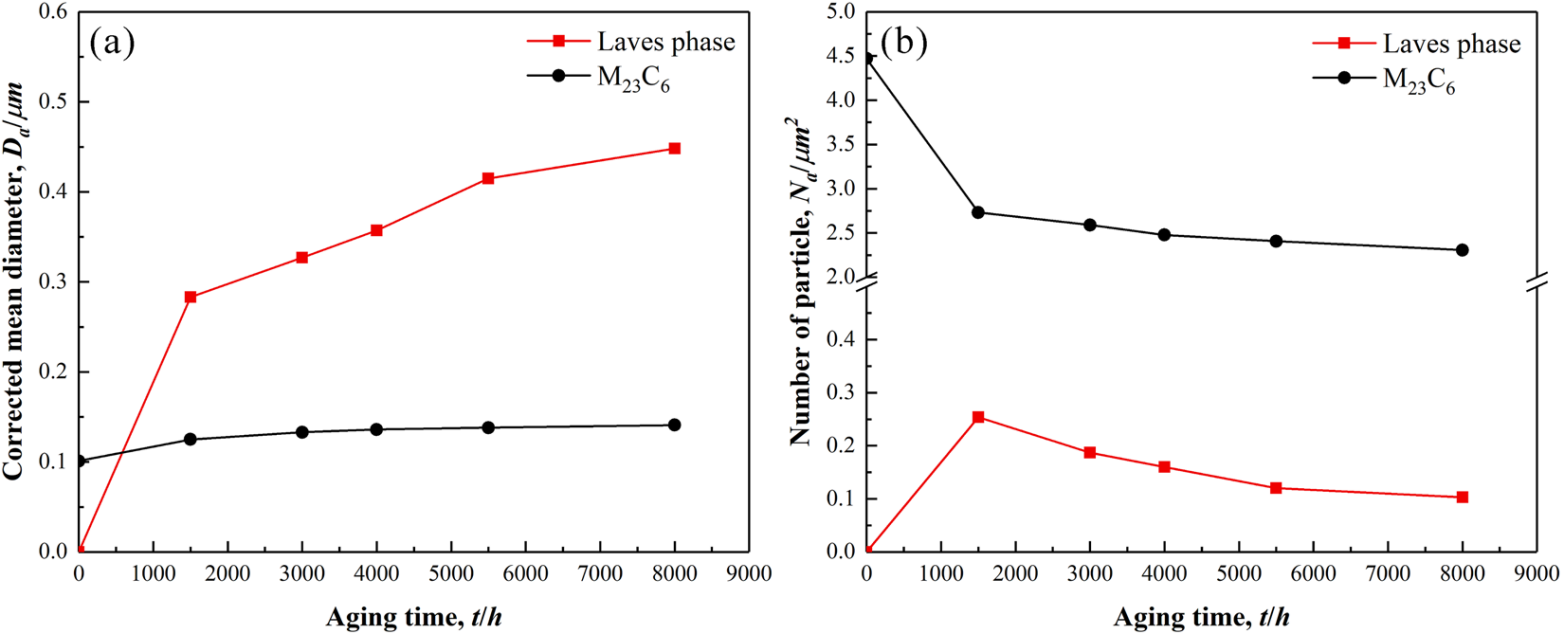
Average diameter of precipitates (**a**) and the number of precipitates per unit area (**b**) of M_23_C_6_ carbides and Laves phases in P92 heat-resistant steels following thermal aging for different times.

**Figure 7:**
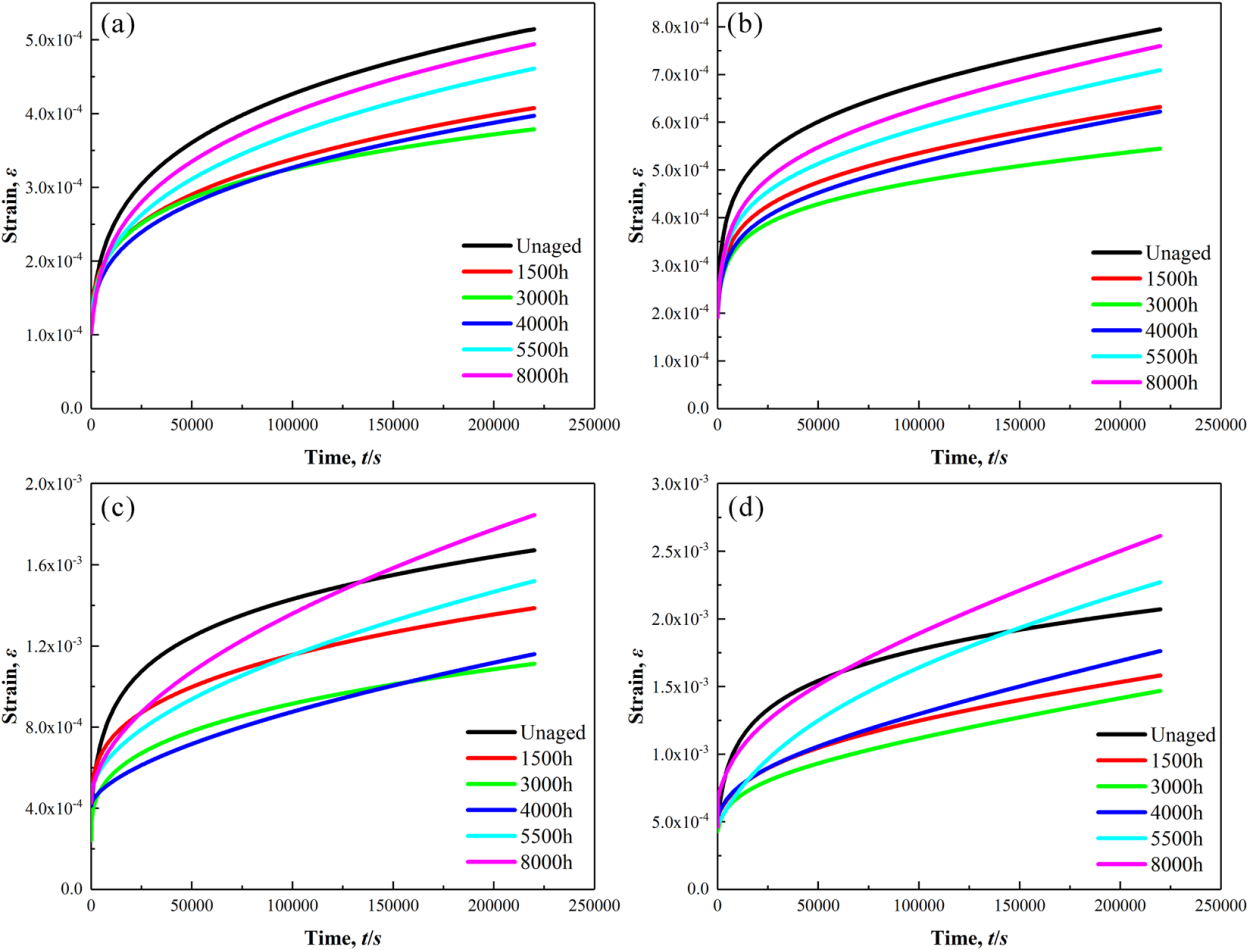
Creep curves of P92 heat-resistant steel specimens following thermal aging for different times at 923 K under stress of 20 MPa (**a**), 35 MPa (**b**), 65 MPa (**c**) and 75 MPa (**d**).

**Figure 8:**
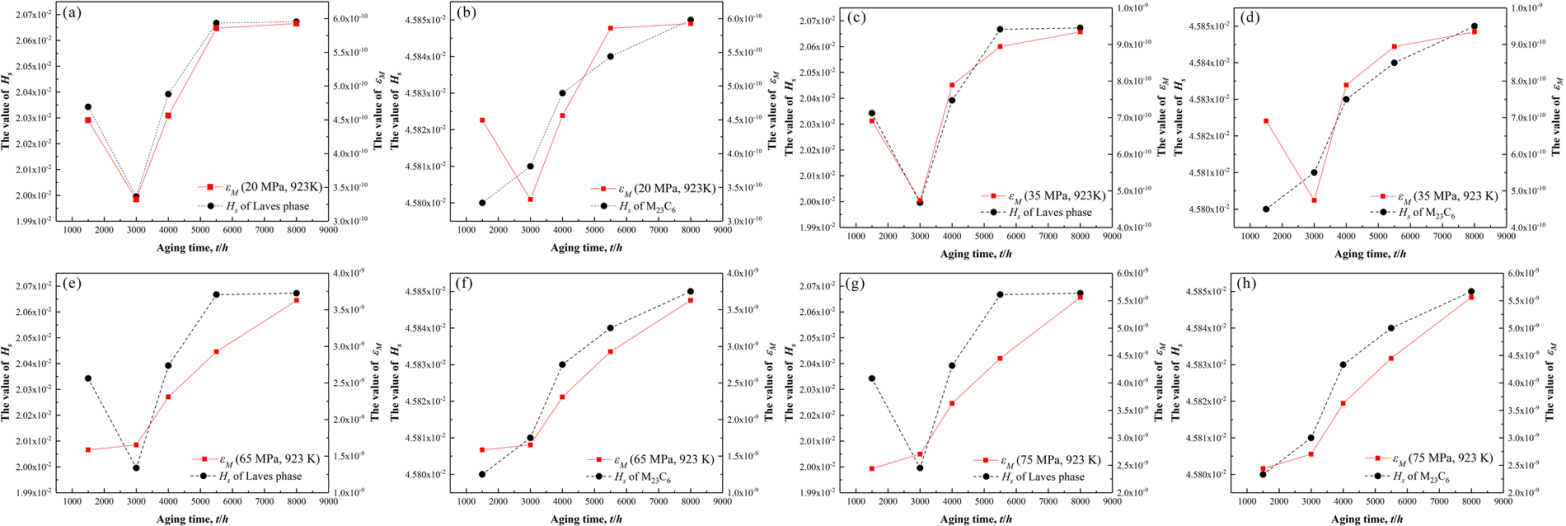
Comparison between the variation trends of *ε*_M_ values for P92 heat-resistant steel at 923 K under different stresses (20 MPa (**a**,**b**), 35 MPa (**c**,**d**), 65 MPa (**e**,**f**) and 75 MPa (**g**,**h**)) and *H*_s_ values for Laves phases and M_23_C_6_ carbides.

